# Intracardiac melanoma metastases on ^18^F-FDG PET-CT—a case report and review of literature with imaging features

**DOI:** 10.1259/bjrcr.20180118

**Published:** 2019-03-28

**Authors:** Shah Sweni, Marianna Fontana, Ana Martinez-Naharro, Malavika Nathan

**Affiliations:** 1 Department of Nuclear Medicine, Royal Free Hospital NHS Foundation Trust, London, United Kingdom; 2 Department of Cardiology, Royal Free Hospital NHS Foundation Trust, London, United Kingdom; 3 Division of Medicine, University College London, London, United Kingdom

## Abstract

Cutaneous malignant melanoma is one of the few malignancies that can metastasize to the heart. It is important not to miss cardiac metastases on imaging as they are often clinically asymptomatic, but if present and undiagnosed could lead to significant cardiac compromise, arrhythmias, congestive heart failure, cardiac tamponade or transient ischaemic attacks.

Identifying intracardiac metastases on imaging often requires a multimodality approach as they can evade detection due to cardiac motion artefact; be confused with intracardiac thrombus, or be misinterpreted as a normal/anatomical finding.

We present an interesting case report of asymptomatic intracardiac melanoma metastases, initially identified on staging 18-fludeoxyglucose positron emission tomography-CT and eventually confirmed on cardiac MRI. The latter was able to differentiate myocardial metastases from tumour thrombus. We also review the relevant literature.

## Background

Cutaneous malignant melanoma, an aggressive neoplasm^1^ with increasing incidence, has a higher propensity to metastasize to the heart compared to other tumours.^2,3^ However, they are rarely detected ante-mortem due to paucity of specific symptoms and signs.^3^

Although, usually present in the context of disseminated metastatic disease, it is important not to miss cardiac metastases on imaging as they are often clinically asymptomatic but if present could lead to significant cardiac compromise in the form of arrhythmias, congestive heart failure, cardiac tamponade, obstruction to the right ventricular outflow and inflow tracts or transient ischaemic attacks. In the past, melanoma patients with cardiac metastases were known to have poor survival rates as they often occurred in conjunction with more disseminated disease. However, due to the recent advent of tyrosine kinase inhibitors and immune checkpoint inhibitors, 5-year survival has improved to 37%.^4^ Nearly 80% of patients with cardiac metastases have BRAF, NRAS or c-Kit mutations, which could potentially extend survival since it allows the administration of targeted therapy.^5,6^ Assessment of the myocardium on melanoma staging imaging is therefore vital to avoid missing cardiac metastases both in the context of disseminated disease or as an isolated finding as both will influence subsequent patient management.

A multimodality imaging approach is often required to diagnose intracardiac metastases as they can evade detection due to the potential pitfalls of each modality used in isolation.

We present an interesting case of asymptomatic intracardiac melanoma metastases that was identified initially on 18-fludeoxyglucose positron emission tomography-CT (^18^F-FDG PET-CT) and subsequently corroborated on cardiac MRI.

We will also discuss the imaging features of intracardiac metastases, with an emphasis on ^18^F-FDG PET-CT to facilitate accurate interpretation by reporting nuclear medicine physicians and radiologists in a timely manner.

## Clinical presentation

A 26-year-old patient, with a positive family history of melanoma and relevant history of occasional sunburn, was diagnosed with a right pre-auricular melanoma, which had arisen from a pre-existing mole. The Breslow depth was 1.56 mm with no ulceration (pathological pT2a /Stage IB). As per NICE guidelines, the patient subsequently underwent a wide local excision and sentinel lymph node biopsy staging. The wide local excision margins and sentinel lymph node biopsy were negative. The patient was followed up clinically on a six monthly basis for the next 3 years.

After 3 years, at a routine follow-up visit, they presented with two asymptomatic but palpable subcutaneous nodules in the left neck and right posterior chest wall respectively. Both lesions were biopsied under ultrasound guidance with histology confirming metastatic melanoma with a positive mutation for BRAF V600E.

**Figure 1.**
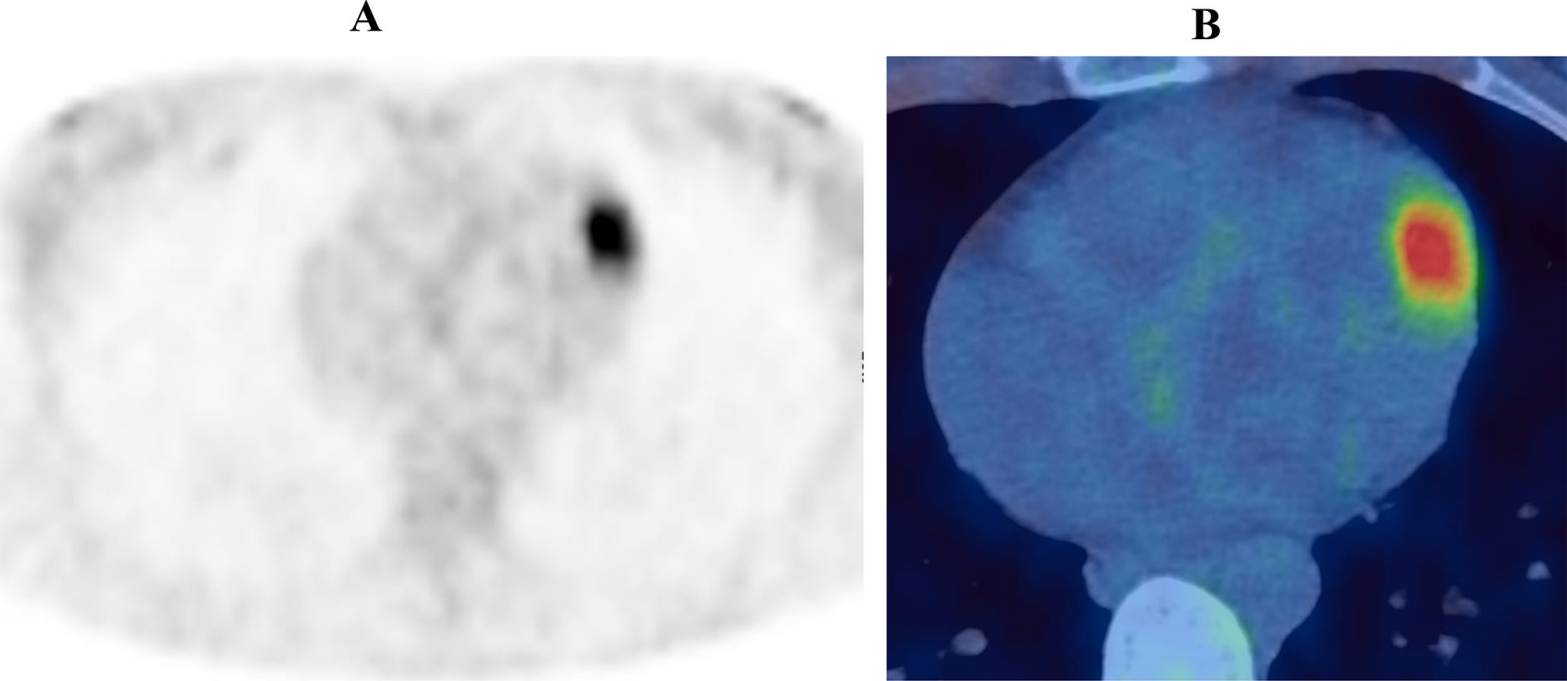
Axial ^18^F-FDG PET only (A) and PET-CT fused (B) images demonstrating the suspicious endocardial (intracardiac) focus of uptake in left ventricle. ^18^F-FDG PET, 18-fludeoxyglucose positron emission tomography.

**Figure 2.**
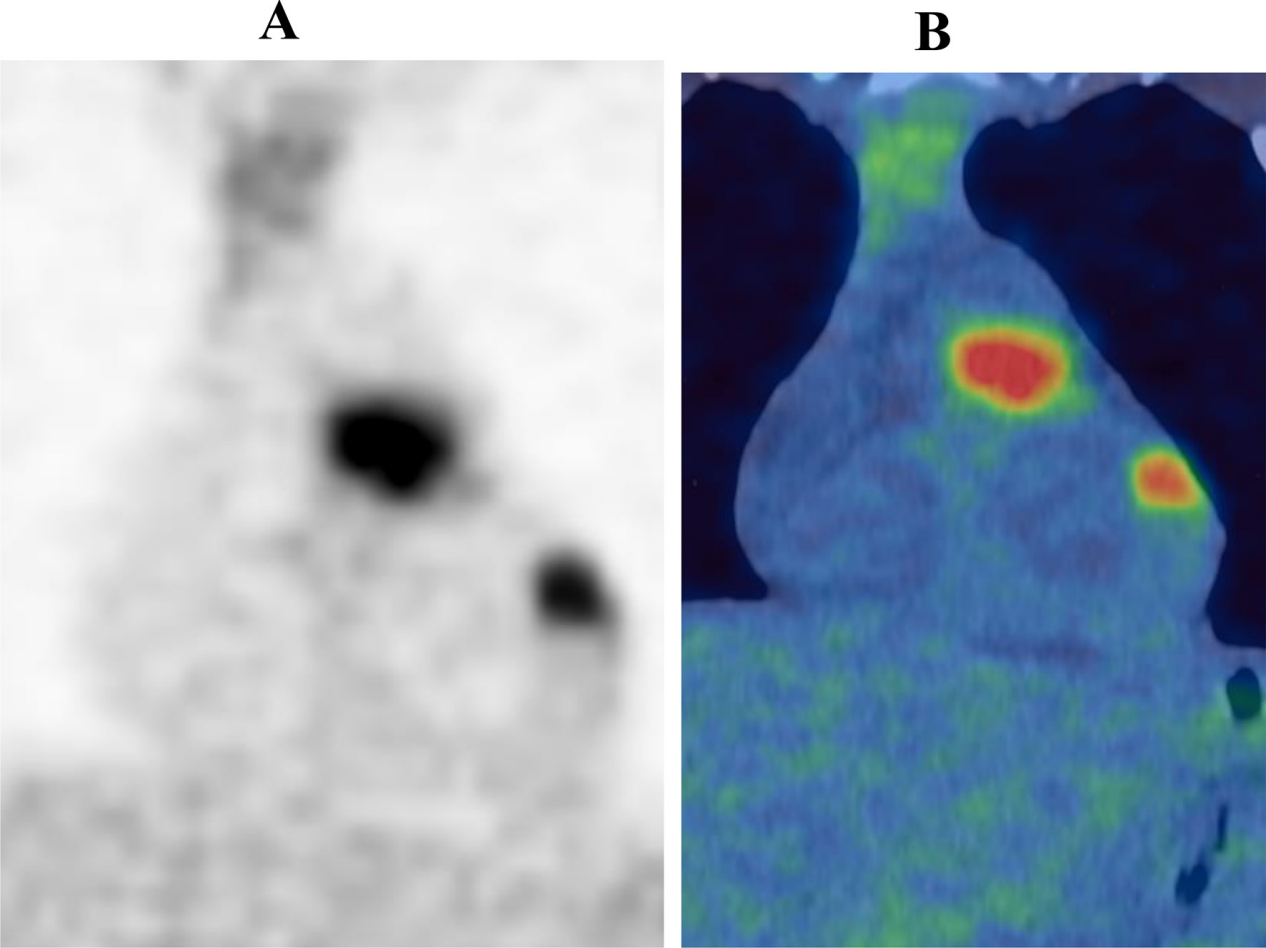
Coronal ^18^F-FDG PET only (A) and PET-CT fused (B) images demonstrating moderate uptake in right ventricular outflow tract lesion and left ventricular lesion. ^18^F-FDG PET, 18-fludeoxyglucose positron emission tomography.

**Figure 3.**
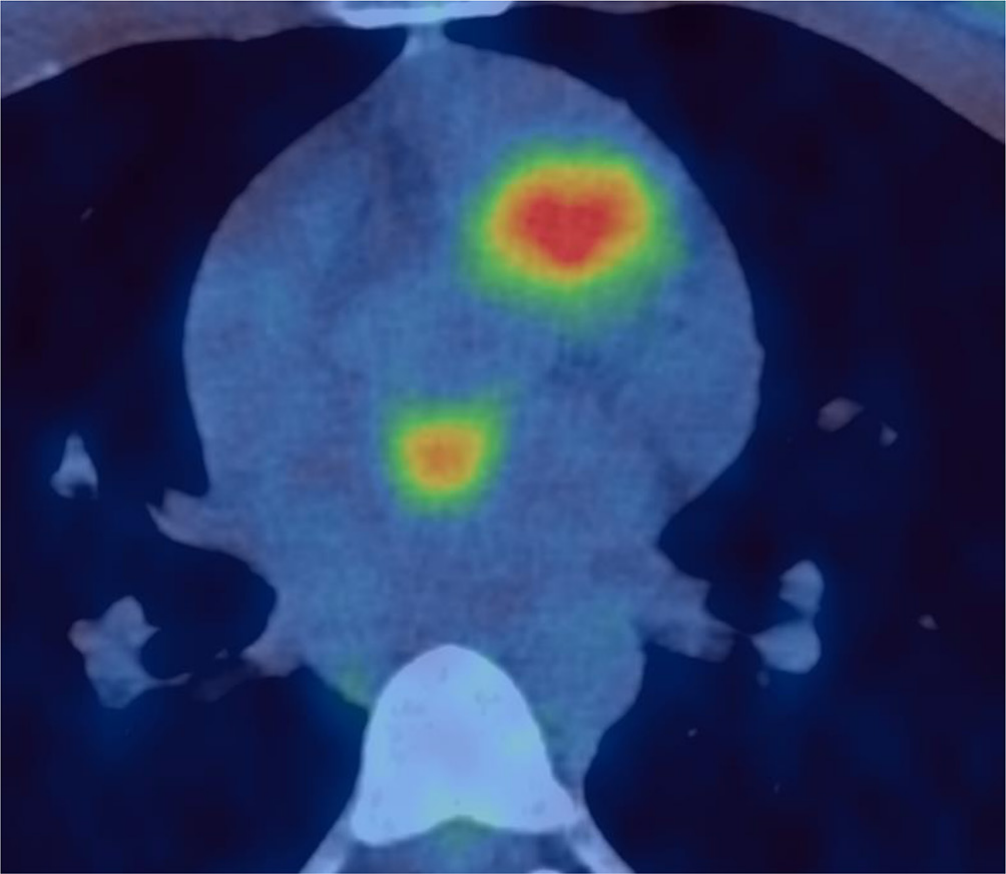
Axial ^18^F-FDG PET-CT fused image demonstrating uptake in the left atrium. ^18^F-FDG PET, 18-fludeoxyglucose positron emission tomography.

The patient underwent a staging ^18^F-FDG PET CT revealing multiple sites of metastatic disease including several suspicious cardiac lesions in the pulmonary trunk/right ventricular outflow tract (RVOT), left ventricular myocardium and left atrium (Figures 1–3).

## Differential diagnosis

The main differentials based on ^18^F-FDG PET CT included either intracardiac myocardial metastatic disease or tumour thrombus. Tumour thrombus in the pulmonary arterial trunk could not be distinguished from RVOT myocardial metastasis on the unenhanced CT component of the FDG PET-CT study.

## Investigations/Imaging

### FDG PET-CT: (Figures 1–3)

PET scan revealed multifocal, intensely avid, metastatic deposits in lower neck, supraclavicular fossa, right apical intercostal region, left lower lobe of the lung and several bilateral pararenal nodules. In addition, focal uptake was seen in the left ventricular myocardium, right pulmonary trunk extending to the right ventricular outflow tract and anterior wall of the left atrium. There was no other organ involvement.

### Cardiac MRI: (Figures 4 and 5)

**Figure 4.**
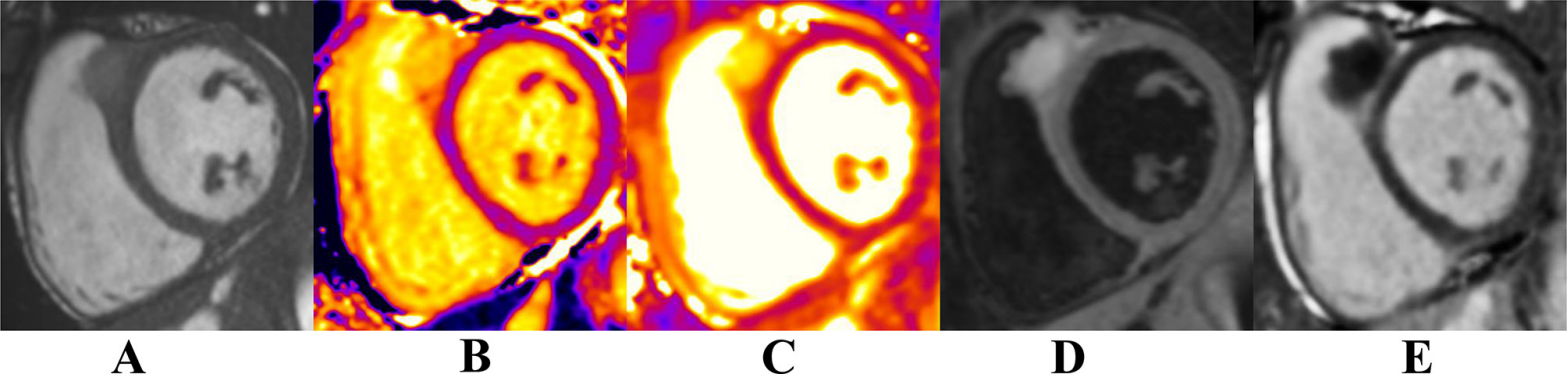
Basal short axis SSFP-cine images (A), corresponding native *T*_1_ mapping (B), *T*_2_mapping (C), *T*_2_weighted imaging (D) and LGE (E). At the level of the RVOT (between the RVOT and LVOT), there is an endocardial nodular lesion measuring 2.6 × 2.2 cm. This lesion has increased values on *T*_1_ and *T*_2_ mapping, increased signal on *T*_2_ weighted imaging and does not enhance on the LGE sequences. LGE, late gadolinium enhancement; LVOT, left ventricular outflow tract; RVOT, right ventricular outflow tract; SSFP, steady-state free precession.

**Figure 5.**
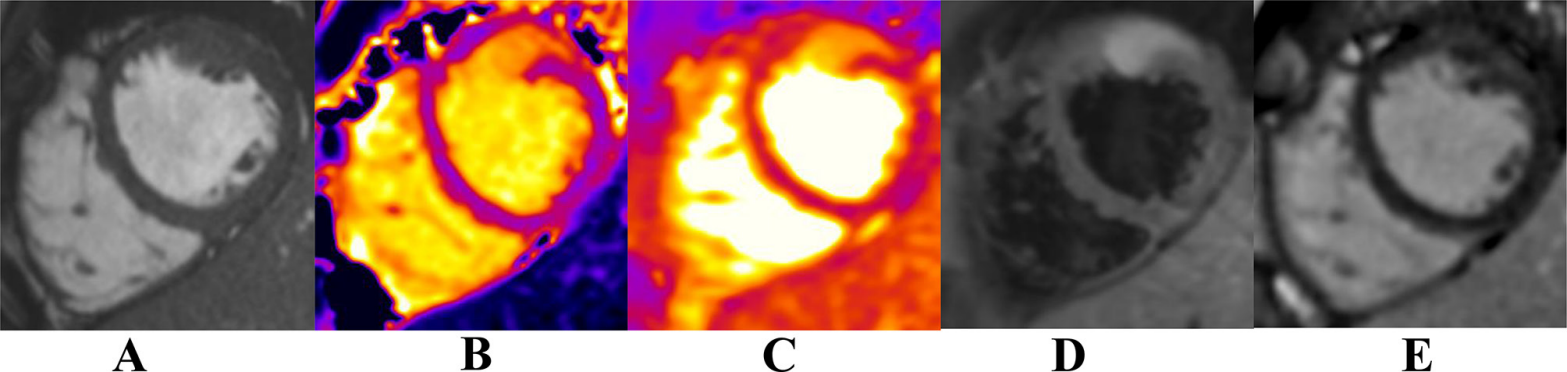
Mid-ventricular short axis SSFP-cine images (A), corresponding *T*_1_ mapping (B), *T*_2_ mapping (C), *T*_2_ weighted imaging (D) and LGE (E). At the level of the mid-anterior wall (adjacent to the anterior papillary muscle), there is an area of heterogenous signal on the cine images. This area has increased *T*_1_ and *T*_2_ values on *T*_1_ and *T*_2_ mapping, increased signal on *T*_2_ weighted imaging and enhances on LGE sequences. LGE, late gadolinium enhancement; SSFP, steady-state free precession.

Cardiac MRI revealed lesions at the level of the mid-anterior wall, RVOT and left atrium. At the level of the RVOT, nodular lesion measuring 2.6 × 2.2 cm showed increased values on *T*_1_ and *T*_2_ mapping and increased signal on *T*_2_ weighted imaging with no enhancement on late gadolinium enhancement (LGE) images. At the level of the mid-anterior wall, adjacent to the anterior papillary muscle, an area of heterogeneous signal was seen on cine images. It showed increased values on *T*_1_ and *T*_2_ mapping and increased signal intensity on *T*_2_ weighted imaging and enhanced on LGE images. The findings are highly suggestive of metastatic melanoma.

## Management

The patient was asymptomatic but was referred urgently to the cardiologists for further assessment. Emergency echocardiography was inconclusive for tumour thrombus and did not demonstrate right ventricular strain. Cardiac MRI (CMR) was preferred to contrast enhanced CT to facilitate differentiation between myocardial metastases and tumour thrombus.

Cardiac MRI confirmed the findings seen on ^18^F-FDG PET-CT with findings highly suggestive of metastatic melanoma.

The patient was commenced on targeted therapy with BRAF tyrosine kinase inhibitors, Dabrafenib and MEK inhibitor, Trametinib.

## Discussion

We have presented an interesting case of intracardiac melanoma metastases initially identified on ^18^F-FDG PET-CT and corroborated on CMR.

Cardiac metastases are rarely diagnosed on routine staging scans for melanoma as they are often asymptomatic and can evade detection due to motion artefacts on CT^2,3^ the modality most commonly used for staging. Cardiac invasion usually occurs due to lymphatic and haematogenous spread and depends on individual aspects affecting histology and functional environment of melanoma cells.^2^

Melanoma metastases may invade any cardiac structure, however, epicardial involvement is most common. Within cardiac chamber metastases, the right heart is more commonly involved (46% right atrium, 18% right ventricle, 18% left atrium).^7^ Myocardial lesions frequently involve the left ventricular free wall and septum, whereas, endocardial metastasis present as intra cavitary lesions. In our case, metastases were identified in the RVOT, left atrium and left ventricle and were intracavitary (endocardial).

Identification of intracardiac metastases can be challenging and often requires a multimodality approach due to the pitfalls of each imaging modality in isolation.

Transthoracic echocardiography is the most frequently used imaging modality for cardiac metastases screening due to its easy availability. In a recent review of cardiac melanoma cases^7^, 82% of cardiac melanoma metastases were identified on transthoracic echocardiography. Trans-oesophageal echocardiography is superior. The shortcomings of echocardiography include restricted ability to distinguish various tissue types and limited acoustic views. However, it is useful for identifying haemodynamic compromise and lesions within the cardiac chambers.^8^

Cardiac metastases can be obscured by cardiac motion artefact on CT.^2,3^ Following intravenous contrast administration, intracardiac metastases can appear hypodense relative to the surrounding myocardium in arterial and portal-venous phase. However, portal-venous phase is better suited, since right heart metastases are prone to dilutional artefact on arterial phase imaging.^2,9^

CMR^10,11^ is ideal for assessment of cardiac masses due to its ability to allow multiplanar reconstruction, detailed tissue characterization and precise anatomical and functional information. It also demonstrates invasion of surrounding structures and depicts better contrast resolution.^12^ On CMR, melanoma metastases have characteristic high signal intensity on both *T*_1_ and *T*_2_ weighted sequences. Gadolinium enhanced sequences helps to distinguish thrombus from tumour.^11,13^

^18^F-FDG PET-CT is known to highlight metastases at unusual or unexpected sites including the heart.^6^ ^18^F-FDG activity patterns of cardiac metastasis of various tumours have been well described^14^, mostly as intensely avid lesions. Various ^18^F-FDG PET-CT imaging manifestations of melanoma metastases to the heart are highlighted in Table 1.

**Table 1.** Various presentations of melanoma metastasis to the heart and imaging features

Age	Gender	Primary melanoma site	Treatment of primary tumor	Cardiac symptoms	Mutation	^18^F-FDG PET-CT	Reference
40	F	Right shoulder	Surgery and interferon therapy	Recurrent syncope and non-sustained VT	BRAF V 600E	Intense avidity in thickened anterior left ventricular wall.	Friedel et.al^15^ .
41	M	Left foot	Recurrent isolated groin lymph node metastasis treated with excision	Asymptomatic	unknown	Intense focal avidity in superior vena cava adjacent to right atrium	Kruger et.al^16^ .
44	F	Occipital	Wide local excision and postero lateral neck dissection + radiotherapy to neck + interferon	Asymptomatic	unknown	Intense FDG involvement at inter atrial region	Tas et.al^11^ .
43	F	Right anterior chest wall	Local resection	Complete heart block, atrial tachycardia	unknown	Focus of markedly increased FDG uptake in proximal and mid-inter ventricular septum	Cheng et. al^17^ .

^18^F-FDG PET, 18-fludeoxyglucose positron emission tomography.

A potential pitfall is that pathological uptake in intracardiac metastases can be obscured by physiological myocardial uptake. Physiological cardiac FDG uptake has been extensively reviewed^12,18^ but we mention it here briefly to understand the important differences compared to cardiac metastases.

Greater than 90% energy requirement of myocardial cells in fasting state, is derived from free fatty acids.^12^ Therefore, oncological PET CT imaging requires careful patient preparation and fasting of minimum 6 h to facilitate free fatty acids metabolism over glucose metabolism. This allows optimal examination by accentuating the differences between normal background tissues (minimal myocardial uptake) and metabolically active neoplasm.^12,13^

Despite fasting, physiological myocardial FDG activity is very variable with a spectrum ranging from being completely absent, focally increased (papillary muscles, atrium, base, anteroapical left ventricle), regionally increased (base and posterolateral left ventricle) to homogenously or heterogeneously diffusely increased.^18^ Even in the same patient the uptake can be spatially and temporally heterogeneous.^19^ The atria are usually non-FDG avid due to minimal energy expenditure. However, focal right atrial uptake can be associated with atrial fibrillation and could be misinterpreted as malignant disease.^18^ Isolated papillary muscle uptake, atrial appendage uptake may also mimic neoplasm or thrombus. Increased right ventricular FDG uptake is known to occur in pulmonary hypertension/right ventricular pressure overload.^18^ Lipomatous hypertrophy of intra atrial septum and pericardial fat may also stimulate metastasis. Moreover, drugs and diet can affect myocardial activity.^12^

Difficulties in diagnosing cardiac metastases could be potentially overcome by special patient preparation or using multimodal approach including cardiac MRI.

### Overcoming pitfalls of physiological cardiac FDG avidity

#### Special patient preparation

Cheng et.al^17^ have demonstrated that special patient preparation with a low carbohydrate, high-fat diet in last 24 h and overnight fasting ensures extremely low background myocardial uptake relative to blood pool activity. This allows metastatic melanoma lesions in the heart to be detected with confidence and can be employed if intra cardiac metastases are suspected clinically or on other imaging modalities. This protocol can be easily implemented/applied routinely in oncology patients with high risk of cardiac metastasis and therefore help identification of these lesions easily.

##### Optimize PET windowing to facilitate cardiac visualization

Upper window level adjusted to facilitate visualization of faint mediastinal nodal and pulmonary nodule activity obscures normal cardiac FDG activity. Separate windowing to optimize evaluation of focal cardiac and para-cardiac FDG findings is needed.

## Conclusion

This case report and literature review highlights the need for Nuclear Medicine physicians and radiologists to be aware of various cardiac manifestations and pitfalls of ^18^F-FDG PET-CT image interpretations in patients with cutaneous metastatic melanoma. Special patient preparation with a low carbohydrate, high-fat diet in last 24 h and overnight fasting could help limit physiological uptake in the heart. Since intracardiac metastasis has implications with respect to management and prognosis of the disease, correct interpretation of the images is vital.

## Learning points

- Intracardiac lesions require a multimodality approach for diagnosis and interpretation.
- Transthoracic echocardiography although has restricted ability to differentiate various tissue types; it can help in identifying haemodynamic compromise.
- Melanoma metastasis demonstrate high signal intensity on *T*_1_ and *T_2_* weighted images on cardiac MRI.
- Adequate patient preparation, slow carbohydrate, high fat diet 24 h prior to imaging and optimizing mediastinal windows helps in distinguishing physiological cardiac activity from pathology on ^18^F-FDG PET-CT.
